# Supporting State Innovation in Alzheimer’s Research

**DOI:** 10.1093/geroni/igaf122.1660

**Published:** 2025-12-31

**Authors:** Joseph Gaugler

**Affiliations:** University of Minnesota, Minneapolis, Minnesota, United States

## Abstract

The State Alzheimer’s Research Support Centers (StARS) is a research support center for state’s dementia care policies. The center is developing and implementing an approach to partnering with a wide range of programs to monitor activities, provide logistical, funding, and coordination support, and establish a robust outreach and dissemination strategy. This presentation will provide an overview of the StARS Centers and examples of state policies and programs that are being evaluated under this initiative.

